# Internet-Delivered Psychological Treatment for Parents With Health Anxiety by Proxy: Replicated Randomized Single-Case Experimental Design

**DOI:** 10.2196/65396

**Published:** 2025-10-02

**Authors:** Katrine Ingeman, Ditte Hoffmann Frydendal, Lisbeth Frostholm, Ellen Bjerre-Nielsen, Kaare Bro Wellnitz, Patrick Onghena, Kristi Wright, Charlotte Ulrikka Rask

**Affiliations:** 1 Department of Child and Adolescent Psychiatry, Research Unit, Aarhus University Hospital Psychiatry, Palle Juul-Jensens Boulevard 175, Aarhus, 8200, Denmark, 45 20542556; 2 Department of Clinical Medicine, Faculty of Health, Aarhus University, Aarhus, Denmark; 3 Department of Functional Disorders, Aarhus University Hospital, Aarhus, Denmark; 4 Faculty of Psychology and Educational Sciences, KU Leuven, Leuven, Belgium; 5 Department of Psychology, Faculty of Arts, University of Regina, Regina, SK, Canada

**Keywords:** health anxiety by proxy, health anxiety, illness anxiety, internet-delivered treatment, single-case experimental design, parental worries, psychological treatment, internet-delivered, internet, parents, anxiety, depression, treatment, severe illness, child, youth, proxy, severe distress, distress, feasibility, anxiety-related symptoms, psychological, mental health

## Abstract

**Background:**

Health anxiety by proxy is characterized by ruminations about severe illness in one’s child that can cause severe distress in affected parents. Health anxiety by proxy may lead to repeated unnecessary medical consultations and checking the child’s body for symptoms, as well as heightened attention to their child’s behavior, sign of illness, and bodily symptoms. It has been hypothesized that health anxiety by proxy may pose a risk for transmission of maladaptive symptom coping and health anxiety from the parent to their child. In spite of this, no targeted treatment has previously been evaluated. Therefore, we developed an internet-delivered psychological treatment containing 8 modules based on cognitive behavioral therapy and acceptance and commitment therapy.

**Objective:**

The objective of this study was to investigate the feasibility and effect of the internet-delivered treatment PROXY for parents with health anxiety by proxy.

**Methods:**

A total of 4 participants with health anxiety by proxy entered a replicated randomized single-case experimental design. They were randomly allocated to a baseline period of 7‐26 days before entering the 8-week treatment and 14‐33 days follow-up phase. The primary outcomes were daily measures of anxiety, impact of anxiety, and value-based actions measured using 5 questions answered on a scale of 1‐10 through a text-message link. The primary outcomes were analyzed using visual analysis and supplemented with statistical randomization tests. Secondary outcomes were standardized questionnaire measures of anxiety-related symptoms, experience of the treatment, and negative effects of the treatment reported using descriptive statistics for each participant individually.

**Results:**

Visual and statistical analyses indicated that PROXY was an effective treatment for 2 participants as the primary outcomes changed in the preferable direction for both of them. The effect of PROXY was questionable for the remaining 2 participants, although visual analysis showed that the impact of anxiety decreased for one of them. The 2 participants with questionable effect also thought that the treatment was too short. All 4 participants were happy with the treatment, but 2 participants experienced that health anxiety for their own health deteriorated during treatment.

**Conclusions:**

PROXY holds potential as a treatment for HA by proxy. However, more work is required to determine when and how PROXY should be introduced to parents with HA by proxy, particularly in relation to duration of treatment, possible comorbidities, and the need for findings to be replicated in larger groups.

## Introduction

Severe health anxiety (HA) is characterized by ruminative thoughts about harboring a serious disease [1]. It is classified as hypochondriasis in the ICD-10 (*International Statistical Classification of Diseases and Related Health Problems, Tenth Revision *[2]) and the *ICD-11* (*International Classification of Diseases, 11th Revision*) [3], or as Illness Anxiety Disorder or Somatic Symptom Disorder in the *DSM-5* (*Diagnostic and Statistical Manual of Mental Disorders *[Fifth Edition] [4]). HA is a debilitating disorder where illness worries are accompanied by a tendency to interpret natural bodily sensations as abnormal or signs of serious illness [5]. Excessive attention directed toward bodily symptoms is common and is typically accompanied by frequent medical examinations, which only provide temporary reassurance [6] and often lead to excessive health care use [7,8]. The disorder is common with a prevalence of 5.7% in the general adult population [9] and 9.5% in the primary sector [1].

Clinical observations and growing evidence suggest that parents can also suffer from HA on behalf of their children [10,11]. This has recently been referred to as HA by proxy [12]. Characteristic features are ruminations about and persistent fear that one’s child has a serious illness that doctors are missing [12]. For some parents, this fear may lead to repeated medical consultations and checking the child’s body for symptoms, as well as heightened attention to their child’s behavior, signs of illness, and bodily symptoms [13,14]. While repeated medical consultations for a child may superficially resemble the clinical presentation of Munchausen syndrome by proxy [12], it is important to distinguish this from HA by proxy. Unlike Munchausen syndrome by proxy—now formally termed factitious disorder imposed on another—where a caregiver intentionally induces or fabricates illness in a child, HA by proxy does not involve deliberate deception or harm.

Research suggests that illness worries and illness behaviors can be transmitted from parents to their children [15-20]. The way parents model coping with their own symptoms [16-18] and how they respond to their children’s health complaints can significantly shape the children’s health attitudes and behaviors [15,21]. For example, a study on pain found that children report experiencing more pain when their parents focus more on their pain symptoms and thereby reinforces the child’s negative illness perception (Levy [22]). Therefore, parental HA by proxy may contribute to the development of maladaptive illness behaviors, perceptions, and health-related worries in the child. In a previous qualitative study, we found that HA by proxy caused distress for the affected parents and impacted their daily life negatively as a result of attempts to control the anxiety or avoid situations that may trigger it [14]. Parents with HA by proxy reported struggling with beliefs that their worrying might be necessary in order to take proper responsibility for their child’s health. At the same time, they realized that their worries led to maladaptive coping behavior, such as asking the child repeatedly about the child's well-being or looking for potential bodily illness signs such as bruises while bathing the child. Also, parents were concerned that their behavior could have negative consequences for their children, for example, that the children over time would develop health worries themselves [14].

To date, research on HA by proxy has been very limited, which means that the condition has not been systematically addressed in health care settings. One of the reasons for this is that until recently, no validated measure existed to address the construct [13,23]. Still, HA by proxy has been suggested as an important treatment target for parents with HA [24]. Currently, the evidence base for cognitive behavioral treatment of HA is strong [25-27]. In particular, acceptance and commitment therapy (ACT) and cognitive behavioral therapy (CBT) have also been investigated in web-based treatment formats with strong positive results for treating HA [28-31]. Given the conceptual and clinical overlap between HA and HA by proxy, internet-delivered treatment of HA by proxy based on these therapeutic approaches may also prove to be effective. However, some parents may only suffer from HA by proxy and not from HA in relation to themselves [11]. Furthermore, distinct cognitive, emotional, and behavioral processes—such as heightened vigilance toward a child’s bodily symptoms, perceived responsibility for the child’s health, and potential intergenerational transmission of HA—are not addressed in regular online HA treatment programs. Therefore, our research group has developed PROXY, the first internet-delivered psychological treatment program specifically designed for HA by proxy [32]. This study aims to conduct an initial assessment of the effect and feasibility of the new internet-delivered psychological treatment program PROXY [32] for parents with HA by proxy.

## Methods

### Design

Single-case experimental design (SCED) was used for this first testing of PROXY as the prevalence of HA by proxy is still unknown. This design allows for detailed, individualized analysis and is well-suited for exploring interventions in emerging or under-researched areas. It entails that (1) the intervention is experimentally manipulated across a series of separate phases and (2) the targeted behavior is measured repeatedly and frequently throughout the phases to investigate whether the changes occur as a function of the manipulated intervention [33].

**Figure 1.**
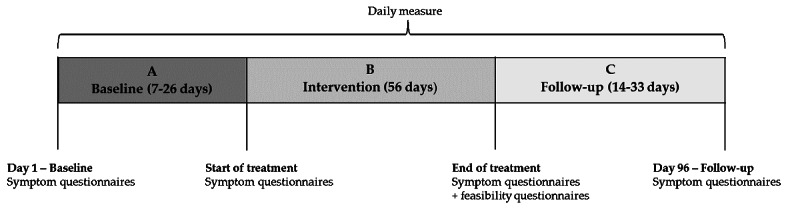
Study design. Adapted from Ingeman et al [32].

This SCED study used a replicated randomized single-case phase design [34], meaning the procedure was replicated with more than a single individual. For each participant, the baseline length was computer-randomized to last between 7 and 26 days before the 8-week treatment period was initiated (56 d). Baseline intervals were stratified into three categories: 7‐11 days, 12‐19 days, and 20‐26 days, with an equal number of participants assigned to each stratum. Follow-up periods ranged from 14 to 33 days, depending on the length of the baseline phase, ensuring that the duration of each individual course was the same. Treatment outcomes were monitored daily by text messages throughout the study providing 96 measurement points in total (according to Kratochwill et al [35] the minimum number of data points are 5 per phase; see Figure 1). Detailed elaborations on design, measures, and intervention content have been reported elsewhere [32].

Daily measure includes 5 questions that participants answered via a link in a daily text message (see Textbox 1). Symptom questionnaires include the Health Anxiety by Proxy Scale, Adult Response to Children’s Symptoms, the Pain Catastrophizing Scale—parent version, and the Whiteley Index 6 Revised. Feasibility questionnaires include the Internet Evaluation and Utility Questionnaire, the Negative Effects Questionnaire—20, and the Experience of Service Questionnaire.

Textbox 1. **Items answered daily.**

How well do the following statements describe your day?

1. I have had persistent worries about my child’s health.
2. I have been worried that my child suffers from a serious physical illness.
3. I have experienced the need to seek reassurance by consulting a doctor, examining my child, searching for symptoms online, or something else.
4. My anxiety for my child’s health has had an impact on my time together with my child.
5. I have done something in the past 24 hours that was important to me in spite of my anxiety.

### Recruitment

Participants were recruited at the Research Clinic for Functional Disorders and Psychosomatics (the Research Clinic) at Aarhus University Hospital, Denmark. The recruitment period was from June 2022 until the end of January 2023.

Participants could self-refer to the treatment by sending a secured email to the Research Clinic. They would then receive a phone call and complete a questionnaire, including the Health Anxiety by Proxy Scale (HAPYS) [23], where the minimum score required to be considered for assessment was 26. This was a pragmatic cut-off score corresponding to the 75% percentile of the HAPYS score in parents without any severe somatic or psychiatric diagnoses. Participants could also be recruited at the research clinic in relation to the routine diagnostic assessments of referred patients for HA where clinical assessment of HA by proxy was included.

The inclusion criteria were: (1) clinically assessed to suffer from HA by proxy, which was defined as being severely impacted by persistent, recurring worries about one’s child’s health, fear that one’s child has a serious illness that is being overlooked, and increased attention to bodily symptoms and physiological reactions that are misinterpreted as signs of serious illness in the child; (2) have children under 18 years without severe diagnoses or disabilities requiring regular hospital visits; and (3) be able to read, write, and speak Danish.

Exclusion criteria were: (1) comorbid diagnoses of substance abuse, bipolar disorder, psychotic disorders (ICD-10: F20-29) or autism spectrum disorders; (2) recently starting up medication for anxiety (within the last 2 months); and (3) suicidal risk.

### Assessment

Clinical assessments were performed by psychologists at the Research Clinic using a short standardized diagnostic interview based on the Schedules for Clinical Assessment in Neuropsychiatry; World Health Organization [36,37] supplemented with assessment of HA by proxy using an interview version of the HAPYS (see section 2.5.2. for a detailed description of the HAPYS; see Ingeman et al [13] and Ingeman et al [23]).

### The Intervention

The internet-delivered treatment program PROXY was developed by our research group and is a new therapist-assisted treatment targeting HA by proxy [32]. It is based on frameworks from CBT and ACT (for details on development procedure and content, see Ingeman et al [32]). PROXY consists of 8 modules that are made available weekly over 8 weeks (the first week, both module 1 and 2 were available). The modules focus on providing psychoeducation about HA by proxy (module 1), identifying personal values (module 2), identifying control and avoidance behavior (module 3), acceptance of unpleasant inner states (module 4), exposure to anxiety-inducing situations (module 5), practicing self-compassion (module 6), seeking support from relatives (module 7), and knowledge about intergenerational transmission of health worries and preparation for continuing on their own and using what they have learned (module 8). The content of each module is presented by text, written exercises, audio files, and patient videos. In this study, the intervention was delivered over the internet in a specialized hospital setting supported by a psychologist who provided written individual feedback through an embedded text message system once a week. Phone calls were made if patients were inactive for more than a week or in need of specific help to move forward in the program.

### Ethical Considerations

All participants received verbal and written information about the project before deciding to participate and gave their written consent to publish data individually and pseudo-anonymized. The participants did not receive any compensation. The project was approved by the Danish research ethics committee (1-10-72-296-20) and registered at the Danish data protection agency (1-16-02-921-17). Furthermore, the project was registered on ClinicalTrials.gov with the US National Library of Medicine on February 04, 2021 (NCT04830605).

### Measures and Data Collection

All data was collected using the online data collection system Redcap [38,39].

### Primary Outcome: Daily Measure

The participants received a daily text message at 7 PM containing a link to 3 items selected from the HAPYS to assess HA by proxy. The selection of these items was based on face validity and an indication of high sensitivity to change [13] (see items 1‐3). The text message also contained an impact question (item 4) and a de novo formulated question about value-based action (item 5). Items were answered on a scale of 1‐10 (1=“does not describe it at all” and 10=“describes it very well”) but coded as 0‐9 for analyses. Scores were summed for items 1‐3 (range 0‐27) while items 4 and 5 were analyzed individually (range 0‐9). For item 5, the scoring was reversed, indicating that for this specific item alone, higher scores corresponded to a greater level of the desired behavior (see Textbox 1).

### Secondary Outcomes

#### Symptom Questionnaires

The symptom questionnaires were answered 4 times during the study: at the beginning of the baseline period, at start of treatment, at end of treatment, and at follow-up except for feasibility measures, which were only answered at the end of treatment (see Figure 1).

*The* HAPYS assessed HA by proxy. It comprises 26 items rated on a 5-point scale (0=Not at all/never, 1=A little/rarely, 2=Some/sometimes, 3=Quite a lot/often and 4=A lot/most of the time; range 0‐104, higher scores indicating more anxiety) and an impact section with additional six items rated on a 4-point rating scale (0=no, 1=yes, a little bit, 2=yes, quite a bit, and 3=yes, a great deal; range 0‐18, higher scores indicating more impact) and two items providing information on time span for worries and if parents perceive their worries to be a problem [13]. In the evaluation of measurement properties, the HAPYS displayed 1-factor dimensionality as well as satisfactory convergent and known-groups validity along with test-retest reliability in a sample of parents with and without HA [23].

The Adult Response to Children’s Symptoms consists of 4 subscales: Protect, Monitor, Minimize, and Distract, which together measure parental behavior when their child is having abdominal pain [40]. For this study, only the protect (13 items) and monitor (4 items) subscales were used, with “abdominal pain” replaced by “feels unwell” to accommodate broader use [24]. Both were scored 0=neverto 4=always (average total score: 0‐4) with higher scores indicating more protective and monitoring parental behavior, respectively. The original ACRS was validated in a sample of parents of children with and without chronic pain. It displays satisfactory convergent and known-groups validity and a 4-factor structure corresponding to the mentioned subscales [41-43].

The Pain Catastrophizing Scale–parent version assesses parents’ thoughts and feelings when their child has pain symptoms on 13 items (0=not at all to 4=extremely [range 0‐52]; higher scores indicating more catastrophizing thoughts) [44]. The questionnaire has demonstrated a 3-factor model with good internal consistency, but the scale is still used with a one sum score [44]. The Danish version showed good face validity after cultural adaptation tested with parents of healthy children [45].

The Whiteley Index 6 Revised (WI-6-R) consists of 6 items to assess illness worries. In contrast to the original Whiteley-7 Index [46,47], WI-6-R excludes items concerning somatic symptoms and includes an item about obsessive illness rumination resulting in strengthened psychometric properties in a population-based sample [48]. Scores range from 0=not at all to 4=a great deal and are summed to a total score of 0‐24 with higher scores representing higher levels of illness worry.

#### Feasibility Questionnaires

The feasibility of PROXY was assessed using three questionnaires that were answered at the end of treatment: (1) The Negative Effects Questionnaire-20 (NEQ), which measured factors related to negative effects of the treatment [49]; (2) the Internet Evaluation and Utility Questionnaire (IEUQ), which was used to evaluate experience of receiving internet-delivered treatment [50]; and (3) The Experience of Service Questionnaire (ESQ), which measures experience with treatment [51,52]. A modified version of the ESQ was used where 7 items were added from the Danish revised version developed by the Department of Psychology and Behavioral Sciences, Aarhus University, Denmark [53]. These additional questions evaluated participants’ experience of how the intervention impacted their family and interaction with their child. A total of 3 questions related to physical settings were removed as they were irrelevant in relation to internet-based treatment. In the present study, all three feasibility measures are assessed and reported at single-item level. Finally, the participants were asked to describe their experience of answering a text message every day.

### Analyses

All graphs for visual analysis and further statistical analyses were performed using Stata 18 (StataCorp) [54]. Demographic characteristics were summarized for each participant individually. Only text messages answered on the same day as they were sent were considered valid and included in the analyses.

Data from the daily measures were submitted to structured visual analysis using the guideline from Lane and Gast [55]. For each participant, daily data was graphed for anxiety level, impact, and value-based action. In total, six main features were visually examined for each graph: (1) level change between phases; (2) variability of data points, both within and between phases (meaning how much the reported values changed and the minimum and maximum value in each phase); (3) trend in data; (4) immediacy of effect*,* which refers to whether the level changed when the intervention was introduced. Specifically, as a delayed effect was expected, we inspected whether the delayed effect was consistent across participants; (5) overlap of data points between phases, that is, the proportion of data points in the intervention phase that were not improved compared to baseline; and (6) consistency of data patterns across participants. The visual analysis was a collaborative effort by the first, second, third, and last authors.

The daily measure of anxiety was also submitted to statistical analyses: first, randomization tests [56-58] were conducted for each participant applying mean comparison of anxiety level before and after treatment entry as test statistic (MeanA–MeanBC=Test-Statistic). Phases B and C were combined because the follow-up phase did not include any new intervention, but still named B and C to indicate end of the treatment. The mean comparison was calculated for every possible randomization scenario for each participant. The *P* values calculated for each participant were combined using the Edgington additive method [59]. Second, the original study protocol indicated that response functions [60] would serve as a secondary randomization test statistic. However, this analysis was omitted due to the very poor fit visually estimated between observed trajectories and hypothesized response function (see Multimedia Appendix 1). Third, effect sizes were calculated for each participant using the nonoverlap method Tau-U, which controls for baseline trends [61]. Finally, data from the additional questionnaires were summarized using descriptive statistics for each participant individually. IEUQ and ESQ were analyzed and reported on item level, and all negative events reported by participants were summarized. Responses provided in free writing were documented as citations.

## Results

### Participants

A participant self-referred but withdrew the consent before clinical assessment. In addition, 2 participants self-referred and 6 patients were recruited at the research clinic. In total, 8 participants were assessed and met the inclusion criteria for participation. However, 3 participants were excluded due to their preference of receiving treatment for HA first or a change of mind regarding participation. Thus, 5 participants entered treatment. A participant dropped out due to technical issues with login (issues were unrelated to the technique at our platform) at the start of treatment, whereas 4 participants completed the treatment and data collection. None of these participants received any additional psychological treatment during their participation in the PROXY program. Table 1 shows the characteristics for the 4 included participants.

**Table 1.** Participant characteristics.

Participants	Age (years)	Sex	Biological children, n	Aged 10 years and younger, n	Education level	Recruitment
Participant 1	33	Male	2	2	>4 y	Clinic
Participant 2	51	Female	2	0	>4 y	Self-referral
Participant 3	34	Female	1	1	Skilled worker	Clinic
Participant 4	31	Female	3	3	>4 y	Self-referral

### Daily measures

A total of 91 (23.7%) daily measures were missing equaling the text messages sent to participants.

### Visual Analysis

#### Daily Anxiety

**Figure 2.**
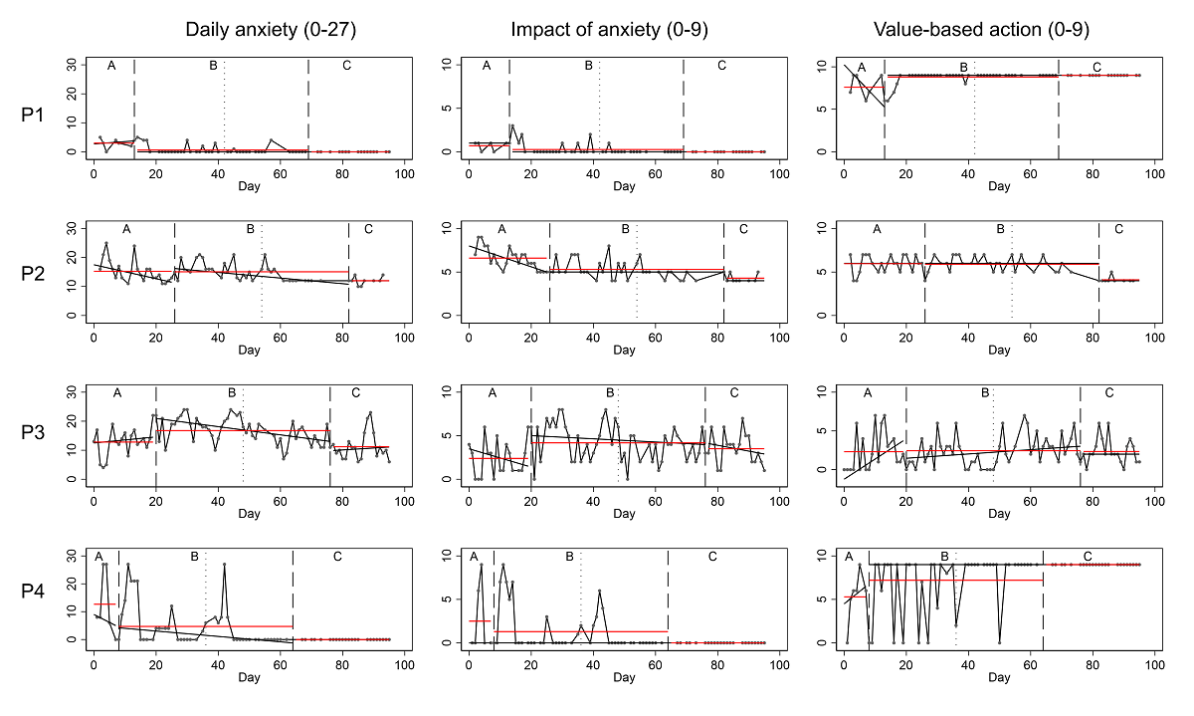
Graphed data of the daily measures for participants 1 to 4 (P1-P4). Red line=mean value; black line=trend line; small vertical dotted line=module 5 that introduces exposure; large vertical dotted line=separates phases; A: baseline phase; B: intervention phase; and C: follow-up phase.

The effect on participants’ anxiety levels did not occur immediately but was delayed. Particularly, toward the end of treatment and during follow-up (see part C in Figure 2), anxiety levels were lowest for all participants (see Figure 2). For Participants 1 and 4, there was a decrease in mean anxiety levels between baseline (see part A) and intervention (part B), and this effect was sustained during follow-up (part C) where anxiety leveled at zero.

For participants 2 and 3, anxiety trend lines were decreasing during intervention (B), but there was no improvement of anxiety mean levels in the intervention period compared to their baseline mean levels (A). However, anxiety mean levels for participant 2 and participant 3 improved during follow-up (C) compared to intervention (B) as illustrated in the graph (see Figure 2) and demonstrated by the mean level changes between phases (see Table 2). These patterns are also reflected in the percentage of overlapping data. From phase A to B, all data points overlapped for participant 2 and participant 3, whereas only 22% and 75% of data points overlapped between phases B and C.

**Table 2.** Mean level change and percent of overlapping data for daily self-reported anxiety.

Participant	Mean level change		POD^a^, %	
	A to B	B to C	A to B	B to C
Participant 1	−2.4	−0.6	19	100
Participant 2	−0.2	−3.1	100	22
Participant 3	4	−5.6	100	75
Participant 4	−8	−4.7	41	100

a POD: Percent of overlapping data. POD is the number of data points above the lowest value of the previous condition [55]. A perfect treatment effect has been achieved if POD equals 0% between phase A and B.

Considerable variability in data points was observed across the participants but also within participant 2, 3, and 4. This was particularly evident for participant 4 where the highest score was 27 and the lowest was zero for both phase A and phase B. The anxiety level was more stable for participant 1 where the lowest score was zero and the highest reported score was 5.

#### Daily Impact of Anxiety and Value-Based Actions

Data patterns for the impact of anxiety followed the patterns of anxiety for participant 1 and participant 4, with great variability for participant 4 and scores leveling at zero toward the end of treatment and during follow-up for both. Participant 1 and participant 4’s reports of value-based actions also stabilized at the most extreme score of 9 towards the end of the study period.

For participant 2, the impact of anxiety improved during baseline (A) and continued to improve through intervention (B) and follow-up (C). Interestingly, this participant reported fewer value-based actions during follow-up (C) compared to baseline (A) and intervention (B).

The variability for impact of anxiety was very high for participant 3, and she did not report experiencing less impact after treatment (see Figure 2). Similarly, this participant did not report more value-based actions during treatment, and the level remained the same between phases, with high variability.

### Statistical Analyses

Effect sizes and randomization tests using mean difference as test statistic are included for each participant in Table 3.

**Table 3.** Results of statistical analyses of daily measures of anxiety.

Participants	*P* value of randomization test^a^ for mean difference	Effect size Tau-U^a^
Participant 1	.45	−0.71
Participant 2	≥.99	−0.01
Participant 3	.35	0.21
Participant 4	.50	−0.61
All combined	.69	—^b^

a Compares baseline to intervention and follow-up combined.

b Not available.

### Additional Questionnaires

There were no missing data on the symptom or the feasibility questionnaires.

### Symptom Questionnaires

Levels of HA by proxy and catastrophizing thoughts decreased for participants 1, 2, and 4 during treatment, while distinct improvements on impact of anxiety measured by the HAPYS were only evident for participant 1 and participant 4. It is less clear if levels of overprotective and monitoring behavior changed significantly for the participants. Levels of own HA were reduced by 50% for participant 4, but were reported unchanged by the remaining participants (see Table 4).

**Table 4.** Descriptive results for symptom questionnaires.

Questionnaire and participants		Time point			
		Baseline	Start^a^	EOT^b^	FU^c^
HA by proxy^d^ 0‐104					
Participant 1		58	51	20	27
Participant 2		74	79	56	48
Participant 3		79	60	74	64
Participant 4		73	72	10	12
Impact of HA by proxy^d^ 0‐18					
Participant 1		9	7	0	0
Participant 2		16	16	12	10
Participant 3		8	8	8	5
Participant 4		10	11	0	0
Overprotectiveness^e^ 0‐4					
Participant 1		1.7	1.0	0.6	0.7
Participant 2		2.7	2.6	2.0	1.8
Participant 3		2.3	2.0	1.5	2.2
Participant 4		2.1	1.8	1.3	2.4
Monitoring behavior^e^ 0‐4					
Participant 1		2.3	1.3	1.0	1.3
Participant 2		3.8	3.8	2.8	3.0
Participant 3		4.0	3.0	2.8	3.0
Participant 4		3.0	2.8	2.8	2.3
Pain catastrophizing thoughts^f^ 0‐52					
Participant 1		24	23	7	8
Participant 2		44	47	29	23
Participant 3		43	49	45	45
Participant 4		27	34	1	0
HA^g^^,h^ 0‐24					
Participant 1		11	18	11	17
Participant 2		19	22	18	22
Participant 3		21	23	24	22
Participant 4		24	24	24	12

a Start: Start of treatment.

b EOT: End of treatment.

c FU: Follow-up after 96 days from study entry.

d Health anxiety by proxy scale.

e Adult response to Child Symptoms—protect and monitor subscales.

f Pain catastrophizing scale-parent version.

g HA: health anxiety.

h Whiteley Index 6 items revised.

### Participant Experience

Based on the ESQ, participants reported to be satisfied with the treatment and the help they had received (see Table 5). Participant 1 was the least satisfied, and participant 4, who had the most significant effect of the treatment, also had the highest rating of PROXY on the ESQ. Citations from the ESQ’s open-ended questions are reported in Multimedia Appendix 2.

**Table 5.** Results from the Experience of Service Questionnaire (answer 0=not true, 1=partly true, and 2=certainly true).

Item	Participant 1	Participant 2	Participant 3	Participant 4
The treatment helped me.	1	2	1	2
If a friend needed this sort of help, I would suggest to them to come here.	2	2	2	2
I have been given enough explanation about the help available here.	2	2	2	2
We feel better in our family now than before the treatment.	1	1	1	2
During the treatment, I became able to change my behavior toward my child in a positive way.	1	1	1	2
During treatment I gained a better understanding of my mental health.	2	1	2	2
I trusted my therapist.	2	2	2	2
The treatment made my child feel worse (reversed item).	0	0	0	0
The treatment made me feel worse (reversed item).	1	0	0	0
It was easy to talk to my therapist.	1	2	2	2
I was treated well by the therapist in charge of my treatment.	2	2	2	2
My views and worries were taken seriously.	2	2	2	2
I feel the people here know how to help me.	1	2	2	2
Overall, the help I have received here is good.	1	2	2	2

All participants reported on the IEUQ that the program was easy to use and that the content was useful and easy to understand. Participants stated that they liked receiving a text message every day and that they did not experience that the daily message affected their answers or their daily lives.

### Negative Events

Negative events that occurred during the treatment period are listed in Multimedia Appendix 3. In free writing, participant 1 stated:

*I think the treatment gave me a better understanding of my anxiety towards my children. But I feel like the treatment has not been good for my own anxiety towards myself. So I would have preferred to have targeted that first*.

[Participant 1]

In addition, participant 2 said:

*I feel like the treatment helped my anxiety towards my children. […] But my anxiety towards my own health has deteriorated.*

[Participant 2]

Participant 3 stated:

*I don’t think that anything affected me negatively. I just think it has been tough but I believe you need to go through these feelings for it to get better.*

[Participant 3]

Furthermore, participant 4 stated:

*I had a flare-up of symptoms during the program. I think it was a result of lack of sleep and stress at home. It gave me more anxiety and a feeling of hopelessness, but I found the right track again. And then the symptoms disappeared.*

[Participant 4]

## Discussion

### Principal Findings

To the best of our knowledge, this paper describes the only existing study exploring treatment specifically targeting HA by proxy. Using SCED, the aim of this study was to conduct an initial assessment of the effect and feasibility of the internet-based treatment program PROXY on parents’ excessive worries about their children’s health and to gather information about individual treatment courses. Overall, the results were not uniform as they indicated that PROXY may be an effective treatment of HA by proxy for two of the participants but questionable for the remaining 2 participants. Furthermore, feasibility results demonstrated general satisfaction with the treatment, but all the participants also reported at least one negative event caused by the treatment. A detailed discussion of the results will be presented in the following sections.

Based on the visual analysis, 2 of the participants (participants 1 and 4) improved on daily measures of anxiety, impact, and value-based action, which was supported by the large effect sizes for participant 1 (*r*=−0.71) and participant 4 (*r*=−0.61) on daily anxiety. This is in contrast to the randomization tests that indicated no treatment effect for either of the participants (*P* values from .35 to 1). This discrepancy might be explained by the choice of test statistics used in the randomization tests, as the mean difference is best suited for testing immediate effect of an intervention [34]. Nevertheless, the inconsistency between the results of the visual analysis and the randomization tests highlights the need for replication of the evaluation of PROXY as a treatment program for HA by proxy.

Based on the visual analysis and the effect size, it appears that participant 2 and participant 3 did not experience an effect of PROXY on their daily anxiety comparing the baseline to the intervention phase. However, participant 2 showed improvements in daily impact of anxiety during the intervention and a small reduction in impact scores on the HAPYS. Within the ACT framework, symptom reduction is not a goal in itself, and the experience of unpleasant inner states is only considered problematic if they impact living, values, and decision-making in a negative way [62]. Following this rationale, it could be argued that P2 did experience some positive effect of PROXY.

During the development of PROXY, we decided to introduce ACT components, such as values and acceptance early in module 2, in order to enhance motivation for performing the exposure exercises [32]. Previous studies have shown large effects of exposure-based CBT for HA [29,30,63]. Thus, in our study, it was expected that the introduction of exposure exercises would have a significant positive impact on participants’ level of anxiety. However, this was not evident from the visual analysis. Exposure is most likely still effective in treating HA by proxy, but the effect might be more gradual. Similar results were found in a SCED study of exposure-based therapy for HA where the specific immediate effects of introducing exposure were also not evident from the visual analyses [64]. In future studies, it may be important to further investigate treatment courses in detail to understand the specific active treatment mechanisms in internet-based anxiety programs like PROXY.

Results from the secondary outcomes measuring symptoms of HA by proxy, catastrophizing thoughts, and overprotective and monitoring behaviors and own HA were also heterogeneous. All four participants improved on the HAPYS (0-104) with between 15 and 61 points. Similarly, patterns of positive change in catastrophizing thoughts were observed during the study period. In fact, the validation study of the HAPYS supported a strong correlation between catastrophizing thoughts and HA by proxy [23]. The present results provide additional support for a relationship between parental HA by proxy and catastrophizing, as they suggest that both seem to be targeted by PROXY. However, it is important to be careful not to generalize the results of these secondary outcomes as they are based on pre-post measures from just 4 participants.

All the participants described that they were happy with the treatment and stated that it had helped them with their distress caused by HA by proxy. However, participant 2 and participant 3 indicated that the treatment period of 8 weeks was too short (see Multimedia Appendix 2). In addition, their daily anxiety level did not improve until follow-up. We aimed for a brief treatment program when deciding for 8 weeks. In comparison, existing internet-delivered treatments for HA all had a duration of 12 weeks [28-31]. Durations of other internet-delivered treatments of anxiety ranged between 5 and 10 weeks [65]. Based on results and feedback from the patients, considerations about the duration of the treatment are important for future use of PROXY.

All participants reported at least one negative event caused by PROXY. Participant 2 in particular reported several negative events, including unpleasant feelings, dependence on treatment, and deterioration of anxiety. Other SCED studies investigating psychological treatment of generalized anxiety disorder and HA also report negative effects [64,66]. In general, temporary distress from psychological interventions is common as a result of confronting distressing thoughts and feelings [67-69]. However, for participant 2, the distress did not seem to be temporary, and she even reported in the NEQ that she was starting to think that her HA by proxy could not be made any better. At the same time, she stated in free text in the ESQ that she felt “seen,” “understood,” and “could recognize almost everything described.” This exemplifies that patients can be satisfied with the treatment and still experience negative events caused by that same treatment. Clinically, we would expect some discomfort of participation in anxiety treatment, because patients often use maladaptive strategies to avoid or control their anxiety. In treatment, these strategies are targeted and altered, for example, through exposure. This will often increase anxiety symptoms temporarily. In accordance with this, an internet treatment of HA found increased anxiety as the most frequent negative effect during treatment; however, there was no association between the experience of negative effects of treatment and symptom deterioration [28]. Overall, this underlines the importance of assessing both negative events and patients' experience of receiving psychological treatment, as patients may be satisfied with treatment, experience effects, and still report negative effects.

Interestingly, both participant 1 and participant 2 stated that their own HA deteriorated during the treatment. Engaging in psychological treatment in general can be quite stressful, and it cannot be excluded that engaging in a treatment where the patients were exposed to illness-related content may have triggered their own HA. In addition, the results seem to indicate that HA and HA by proxy could be seen as two separate anxiety conditions with symptoms that do not follow each other or respond equally to treatment. Furthermore, it raises important considerations regarding when and how to introduce PROXY when parents suffer from both HA and HA by proxy.

### Strengths and Limitations

The reporting of this study followed the recommendation of the Single-Case Reporting Guideline In BEhavioural Interventions statement [70] and has a number of strengths. First, the visual analysis was supplemented by statistical analyses. Statistical analyses in SCED research have previously been considered controversial [55], but in recent years, several researchers have advocated for the supplement of statistical analyses [71-73]. Second, randomizing the start of the intervention—and thereby, the length of the baseline phase—for each participant is a key strength of the study, as it enhances internal validity [56,58]. Third, the combination of daily measures, feasibility measures, and questionnaires to obtain information on participants’ experience as well as effect of PROXY was a considerable strength of the study, and finally, the participants underwent systematic and diagnostic assessments for other mental health disorders before inclusion in the project.

There are also some methodological limitations that warrant consideration. The first concerns the recruitment strategy, which entailed recruitment of participants who also suffered from HA. This overlap between HA and HA by proxy complicates the attribution of outcomes specifically to HA by proxy, as it is unclear whether observed changes (including the deterioration in own HA reported by two participants) reflect effects of the intervention on HA by proxy, on general HA, or on the interaction between the two. It is also possible that participants with high levels of own HA may have had less motivation or ability to focus on the PROXY treatment. Future studies with larger samples and stratified inclusion are needed to disentangle treatment effects on HA by proxy from those on general HA. The second limitation concerns the causal relationship between the changes we see in the patients’ anxiety level and the intervention. Confounding factors that are time-related can be statistically controlled for by randomly determining the intervention start point (the baseline length) in a SCED [35]. It remains unknown whether the baseline in the present study was long enough to conclude that the changes seen were an effect of the intervention and not due to maturation or spontaneous improvements in symptoms, natural fluctuations, or other factors. In the present study, the participants were randomized to one of 20 possible treatment entries. If the observed value from the test statistic was the single most extreme value obtained out of all the randomization possibilities, the *P* value would be 1/20=.05. This means that in our study, the smallest possible *P* value was .05. Therefore, randomization tests require a large amount of randomization possibilities to obtain a small *P* value. This means that when applying the method to SCED for determining baseline lengths, it also becomes an ethical question of how long participants need to wait before they enter the treatment, and for how long it is feasible to obtain daily measures. In our study, the percentage of missing data was already at 23.7% which must be considered a limitation in itself. Further limitations pertain to the reliability and validity of the visual analysis of the daily measures. In general, researchers agree on the six features assessed in this study [74,75]. Nevertheless, visual analysis inherently involves an element of subjectivity. Some researchers argue that the key to visual analysis is to maintain structure [74,76]. Other researchers attempt to remove subjective influence by incorporating statistical calculations and rules in the visual analysis (eg, 55,75,77). A final limitation is the fact that the daily measures relied on self-report, which has been raised by several researchers as a potential issue in SCED studies because the validity of daily self-report measures has often not been investigated and because of the risk of response bias like social desirability [78-84]. The inclusion of clinician-based evaluations of anxiety levels, in addition to self-reports, would have strengthened the assessment of the primary outcome. Yet, the use of self-report measures in SCED studies of interventions for anxiety disorders is not uncommon [66,81,85]. In our study, the most suitable method for assessing HA by proxy was through self-report measures as we were interested in exploring the subjective experience of parents’ daily anxiety level, impact of anxiety, and value-based actions. Summing up, when replicating the SCED study, it is important to consider how to strengthen the validity and reliability of the daily self-report measures; to supplement the visual analysis with further statistical analyses; and to increase the internal validity of the study. The latter can be achieved, for example, by having participants receive treatment concurrently in time and by extending the maximum baseline length [33].

### Conclusions and Future Research Perspectives

To the best of our knowledge, PROXY is currently the only existing treatment for HA by proxy. While findings from this initial study are mixed, they suggest that the intervention may be feasible. Key questions remain regarding effectiveness, optimal treatment duration, timing, and its use alongside treatment for primary HA. Future research on treatment of HA by proxy should focus on recruiting a larger sample and introducing methodological refinements to enable stronger conclusions about treatment effects.

## Supplementary material

10.2196/65396 Multimedia Appendix 1 Response function for participants in PROXY.

10.2196/65396 Multimedia Appendix 2 Experience of Service Questionnaire answers to open-ended questions at the end of treatment.

10.2196/65396 Multimedia Appendix 3 List of reported negative events at the end of treatment. Participants 1, 2, 3, and 4 indicate the participants who reported having experienced the particular event.

## Abbreviations

ACT: acceptance and commitment therapy
CBT: cognitive behavioral therapy
*DSM-5*: *Diagnostic and Statistical Manual of Mental Disorders (Fifth Edition)*
ESQ: Experience of Service Questionnaire
HA: health anxiety
HAPYS: Health Anxiety by Proxy Scale
*ICD-10*: *International Statistical Classification of Diseases and Related Health Problems, Tenth Revision*
*ICD-11*: *International Classification of Diseases, 11th Revision*
IEUQ: Internet Evaluation and Utility Questionnaire
NEQ: Negative Effects Questionnaire-20
SCED: single-case experimental design
WI-6-R: Whiteley Index 6 Revised
