# Supplementary figures and images for: Internet-Delivered Psychological Treatment for Parents With Health Anxiety by Proxy: Replicated Randomized Single-Case Experimental Design
Source: JMIR Form Res. 2025 Oct 2;9:e65396. doi: 10.2196/65396 (PMC12490778; doi:10.2196/65396)

Response function for participants in PROXY.


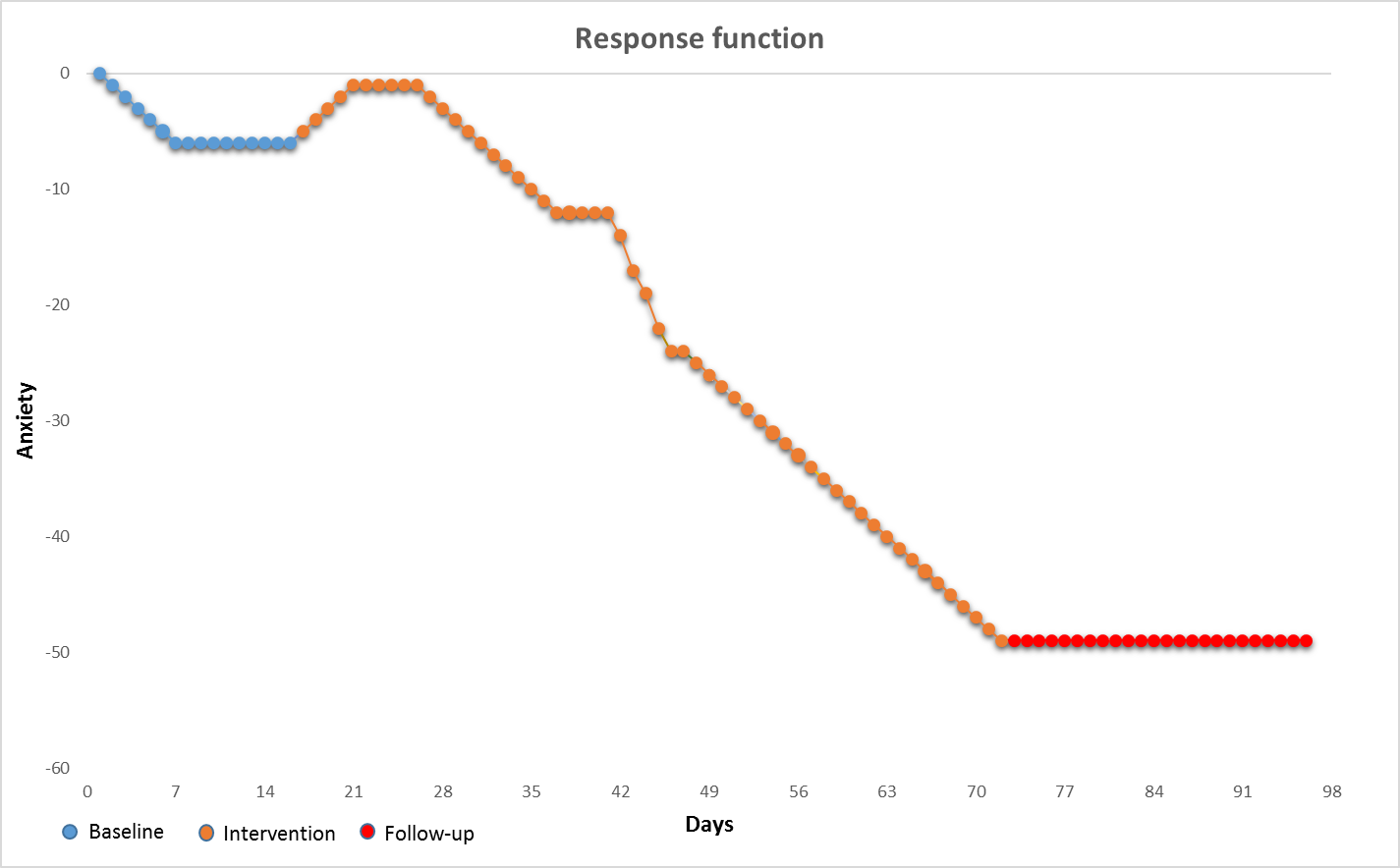

Supplement: Multimedia Appendix 1 [file formative-v9-e65396-s001.docx]
