# Supplementary material for: Internet-Delivered Psychological Treatment for Parents With Health Anxiety by Proxy: Replicated Randomized Single-Case Experimental Design
Source: JMIR Form Res. 2025 Oct 2;9:e65396. doi: 10.2196/65396 (PMC12490778; doi:10.2196/65396)
Supplement: Multimedia Appendix 2 [file formative-v9-e65396-s002.docx]

| Experience of Service Questionnaire answers to open-ended questions at the end of treatment. | | |
| --- | --- | --- |
|  | What do you think was good about the treatment? | Was there anything you didn't like or that could be improved? |
| P1 | *The treatment has helped me understand my anxiety for my children and that it may not be as severe as I might have thought..* | *Online communication doesn’t work very well for me, and it’s difficult to take phone calls because you are in the middle of a busy workday. Perhaps a text message system would be better instead of having to log in to the computer every time.* |
| P2 | *To be seen and understood. What have taken up so much of my mental space has a name. I could recognize almost everything described.* | *I feel like it is difficult to "let go" of the program. I need support, so maybe the treatment could last longer? Or a follow-up at later time to see how the exercises are going.* |
| P3 | *To have some tools I can use to work on myself. That I do not feel quite as alone anymore (because there were videos of others telling their stories).* | *Perhaps I don’t think 8 weeks is enough. I had a feeling that everyday life was somewhat challenging it. But then again, maybe it’s difficult for me to say because I’m just now realizing what I need to work with from the program.* |
| P4 | *I’ve been freed from my health anxiety on behalf of my child, which is a huge relief. I’m very grateful for this program. Sometimes the thoughts appear, but I’m able to let them pass.* | *No. I am very grateful.* |
