# Supplementary material for: Internet-Delivered Psychological Treatment for Parents With Health Anxiety by Proxy: Replicated Randomized Single-Case Experimental Design
Source: JMIR Form Res. 2025 Oct 2;9:e65396. doi: 10.2196/65396 (PMC12490778; doi:10.2196/65396)
Supplement: Multimedia Appendix 3 [file formative-v9-e65396-s003.docx]

Table S1. List of reported negative events at the end of treatment. Participants 1, P2, P3, and P4 indicate the participants who reported having experienced the particular event.

| Experienced negative event | Caused by treatment | Caused by other circumstances | Total |
| --- | --- | --- | --- |
| I felt like I was under more stress. | Participant 1 | Participant 2 + Participant 3 | 3 |
| That is, experienced more anxiety. | — | Participant 2 + Participant 3 + Participant 4 | 3 |
| I felt more worried. | — | Participant 1 + Participant 2 + Participant 4 | 3 |
| That is, experienced more hopelessness. | — | Participant 4 | 1 |
| That is, experienced more unpleasant feelings. | Participant 2 | Participant 3 | 2 |
| I felt that the issue I was looking for help with got worse. | Participant 2 | — | 1 |
| Unpleasant memories resurfaced. | Participant 2 + Participant 3 + Participant 4 | — | 3 |
| I started thinking that the issue I was seeking help for could not be made any better. | Participant 2 | — | 1 |
| I think that I have developed a dependency on my treatment. | Participant 2 | — | 1 |
| I did not always understand my treatment. | Participant 2 | — | 1 |
| Total | 9 | 10 | 19 |
